# Genomic and transcriptomic analysis of the AP2/ERF superfamily in *Vitis vinifera*

**DOI:** 10.1186/1471-2164-11-719

**Published:** 2010-12-20

**Authors:** Francesco Licausi, Federico M Giorgi, Sara Zenoni, Fabio Osti, Mario Pezzotti, Pierdomenico Perata

**Affiliations:** 1 Plant Lab, Scuola Superiore Sant'Anna, Piazza Martiri della Libertà 33, 56127 Pisa, Italy; 2 Max Planck Institute of Molecular Plant Physiology, Am Mühlenberg 1, 14476 Golm, Germany; 3 Dept Sciences, Technologies & Markets for Grapevine & Wine, University of Verona, I-37029 San Floriano Di Valpolicella, VR Italy; 4 IBIMET-CNR, Via P. Gobetti 101, 40129, Bologna, Italy

## Abstract

**Background:**

The AP2/ERF protein family contains transcription factors that play a crucial role in plant growth and development and in response to biotic and abiotic stress conditions in plants. Grapevine (*Vitis vinifera*) is the only woody crop whose genome has been fully sequenced. So far, no detailed expression profile of AP2/ERF-like genes is available for grapevine.

**Results:**

An exhaustive search for AP2/ERF genes was carried out on the *Vitis vinifera *genome and their expression profile was analyzed by Real-Time quantitative PCR (qRT-PCR) in different vegetative and reproductive tissues and under two different ripening stages.

One hundred and forty nine sequences, containing at least one ERF domain, were identified. Specific clusters within the AP2 and ERF families showed conserved expression patterns reminiscent of other species and grapevine specific trends related to berry ripening. Moreover, putative targets of group IX ERFs were identified by co-expression and protein similarity comparisons.

**Conclusions:**

The grapevine genome contains an amount of AP2/ERF genes comparable to that of other dicot species analyzed so far. We observed an increase in the size of specific groups within the ERF family, probably due to recent duplication events. Expression analyses in different aerial tissues display common features previously described in other plant systems and introduce possible new roles for members of some ERF groups during fruit ripening. The presented analysis of AP2/ERF genes in grapevine provides the bases for studying the molecular regulation of berry development and the ripening process.

## Background

The AP2/ERF superfamily is one of the largest groups of transcription factors in plants [1]. It includes all genes coding for at least one APETALA2 (AP2) domain and can be further separated into the ETHYLENE RESPONSE FACTOR (ERF), the AP2, and the RAV families. The AP2 domain, which is responsible for DNA binding, was named after the *Arabidopsis thaliana *APETALA2 protein identified by Jofuku *et al*. [2]. Subsequently, tobacco (*Nicotiana tabacum*) transcription factors containing a motif related to the AP2 domain were also identified [3]. Since these proteins are able to bind an ethylene responsive DNA element (AGCCGCC), they were classified as ERFs (Ethylene Responsive Factors) and their DNA binding domain was named after them [4].

The subdivision of the AP2/ERF group into families is based on the number of AP2 domains present in the proteins, together with the presence of other DNA binding domains. The AP2 family contains proteins with a double, tandem repeated, AP2 domain [5]. The ERF family genes code for transcription factors with a single AP2 domain. Lastly, the RAV family encodes proteins possessing a single AP2 domain plus an additional B3 domain, which is also present in other, non-ERF transcription factors [6].

Two major efforts to define a nomenclature for ERF sequences have been published. Sakuma *et al. *[7] divided the *Arabidopsis *ERF family into two subfamilies based on the amino acid sequence of the DNA binding domain: the CBF/DREB subfamily (group A) and the ERF-like subfamily (group B), both further subdivided in six groups. Nakano *et al*. [8] on the other hand, proposed an innovative classification for *Arabidopsis *ERFs, based on the amino acid sequence of the whole proteins, splitting the family into ten groups.

Despite the relatively high sequence conservation of the AP2/ERF domain, the number of DNA elements bound by different AP2/ERF transcription factors is extremely wide [[3,9] and [10]].

The DREB group interacts with the core sequence CCGAC [11], while the ERF group typically binds to an AGCCGCC sequence, called GCC box [3].

In the AP2 family, a binding element gCAC(A/G)N(A/T)TcCC(a/g)ANG(c/t) has been reported for the protein AINTEGUMENTA (ANT) [12], and the same sequence was shown to be bound by five other AP2 members [13]. This DNA element is quite distinct from the consensus sequence CCGA/CC bound by the DREB/ERF group and, surprisingly, is not composed of two similar half sites, since the AP2 proteins contain a double AP2 domain [14].

AP2/ERF transcription factors regulate a number of biological processes including development, reproduction, responses to hormones, adaptation to biotic and abiotic stresses [15-18] and [19].

After the release of the whole genomic sequences of several plant organisms, including Arabidopsis, rice (*Oryza sativa) *and poplar (*Populus tricocarpa*), the AP2/ERF transcription factor superfamily was analyzed, both to place each member in an organized nomenclature system, and to provide maps of their expression. To date, two full genome sequences of highly homozygous and heterozygous grapevine (*Vitis vinifera*) Pinot noir genotypes have been carried out [[20,21] and [22]]. These two milestones provided a useful genomic platform to study this fruit crop. As AP2/ERF transcription factors are involved in flower development and tolerance to biotic and abiotic stresses, their superfamily represents one of the best pools to investigate, when searching for important grapevine traits. Since genome scale analyses of the transcriptional response to the development and environmental stimuli require a precise and complete annotation in order to provide reliable and exhaustive data, we decided to annotate the ERF family members and create a qRT-PCR platform that allows investigating their expression profile.

A recent study suggested that 132 genes encoding AP2/ERF proteins are present in the grapevine genome [23]. However, a higher number of AP2/ERF genes is present in Arabidopsis and poplar genomes (147 and 202 sequences, respectively). We therefore re-screened the grapevine genome for AP2/ERF sequences, adopting two different strategies, in order to accurately identify AP2/ERF-like sequences in the *Vitis vinifera *genome. In this study we provide a characterization of the grapevine AP2/ERF transcription factor superfamily and demonstrate that it is in fact composed of almost 149 genes. Moreover, using qRT-PCR platform encompassing the whole ERF/AP2 superfamily, we show how AP2/ERF-like genes are expressed in both vegetative and reproductive tissues at different developmental stages, and we infer roles and putative targets for some of these genes. Overall, our analysis suggests that AP2/ERF proteins play a strong role in ripening-related processes.

## Results

### Identification of the AP2/ERF family transcription factors in *Vitis vinifera*

As result of an extensive search for AP2-domain containing proteins, 149 distinct AP2/ERF putative TFs were identified (Additional file 1, Table S1). One hundred and twenty two genes encoding for proteins with a single AP2/ERF domain were assigned to the ERF superfamily. The AP2 family was grouped into 20 genes, that could be identified due to the tandem repeated double AP2/ERF motif. Six genes, containing a single AP2/ERF DNA binding domain together with a B3 type domain, were assigned to the RAV family. A single AP2 protein, GIDVvP00018355001, showed a low similarity to the other ERF sequences although it is homologous to the Arabidopsis ERF transcription factor At4g13040. A similar gene has also been identified in *P. trichocarpa *and named Soloist [24]. Surprisingly, the *Vitis *gene annotated as Soloist in the grapevine genome by Zhuang *et al*. [23] is not homologous to the poplar Soloist. Zhuang *et al*. [23] named protein sequence GSVIVP00025602001 as Soloist, although its similarity to the sequences of Arabidopsis At4g13040 and PtSoloist is rather low. On the other hand, GIDVvP00018355001 is the closest homolog of both At4g13040 and PtSoloist in the grapevine genome (Additional file 2, Figure S2).

Previous annotations of AP2/ERF genes in poplar [24] and grapevine [23] followed the nomenclature proposed by Sakuma *et al*. [7], based on a homology of the DNA binding domain alone. However, Nakano *et al*. [8] proposed an alternative method, based on the presence of domains that were different from the DNA binding domain. Therefore we subdivided the *grapevine *ERF genes into 11 groups, according to their similarity to the Arabidopsis ERF sequences. Amino acid motifs located outside the DNA binding domain are conserved among the *Arabidopsis *[8] and *Vitis *ERF proteins (Additional file 3, Table S3).

Taken as a whole, the ERF/AP2 superfamily has a similar number of genes in grapevine (149) and *Arabidopsis *(147), while it is bigger in poplar (202) and rice (180). The number of RAV genes is highly conserved among species with six members in dicots and five genes in rice. The AP2 family encompasses a similar number of genes in *V. Vinifera *(20) and *A. Thaliana *(19), while this increases to 26 in poplar and 29 in rice (Table 1). The Soloist protein, coded by a single-copy gene and characterized by a low conservation at the ERF DNA-binding domain, was present in all the plant genomes considered. Although the overall number of sequences belonging to the ERF family was conserved in grapevine and *Arabidopsis*, noteworthy differences existed between groups (Table 1). Group III, VIII and X had a very similar number of genes (*Vitis *to *Arabidopsis *ratio lower than 1.25 or greater than 0.7). On the other hand, groups II, IV, VI, VII, and VI-L in *Vitis vinifera *contained half the number of members than *Arabidopsis *(Table 1). The opposite trend was observed for groups V and IX, where the number of members was more than double compared to *Arabidopsis*. Interestingly, in the poplar genome, group IX and V genes were also more than the twice their *Arabidopsis *counterparts. The poplar genome appeared to have a higher number of ERF genes in most of the groups. ERF proteins belonging to the Xb-like group were not found in the grapevine genome.

**Table 1:** A comparison of AP2/ERF families and groups between monocot (Oryza sativa) and dicot (Arabidopsis thaliana, Populus trichocarpa, Vitis vinifera) species.

Family	Group	*Arabidopsis*	*Vitis*	*Poplar*	*Rice*	RATIOS		
						*Vitis*/*Arabidopsis*	*Vitis*/*Poplar*	*Vitis*/*Rice*
**ERF**	**I**	10	5	5	9	0.50	1.00	0.56
	**II**	15	8	20	16	0.53	0.40	0.50
	**III**	23	22	35	27	0.95	0.62	0.81
	**IV**	9	5	6	6	0.56	0.83	0.83
	**V**	5	11	10	8	2.20	1.10	1.38
	**VI**	8	5	11	6	0.63	0.45	0.83
	**VII**	5	3	6	15	0.60	0.50	0.20
	**VIII**	15	11	17	15	0.73	0.65	0.73
	**IX**	17	40	42	18	2.35	0.95	2.22
	**X**	8	10	9	12	1.25	1.11	0.83
	**VI-L**	4	2	4	3	0.50	0.50	0.67
	**Xb-L**	3	0	4	10			
		122	122	169	145	1.00	0.72	0.84
**RAV**		6	6	6	5	1.00	1.00	1.20
**AP2**		18	20	26	29	1.11	0.77	0.69
	**Soloist**	1	1	1	1	1.00	1.00	1.00
		147	149	202	180	1.01	0.73	0.83

Number of genes present in each family and group within the AP2/ERF superfamily in the plant species whose genome has been fully sequenced. A comparison of group and family-size between the grapevine AP2/ERF sequences identified in this study and those present in the Rice, Poplar, Arabidopsis Transcription Factor Databases are provided as ratios.

### Accuracy of protein predictions

Proteins sequences from the Genoscope database have been deduced using an automated method [25]. However, this methodology is prone to errors [26]. We found similar issues in grapevine predictions. In fact, some gene annotations and intron junctions did not match those of the Arabidopsis and poplar homologs. We found unrealistic introns of 8.4 and 35 Kb predicted in *GSVIVP00009519001 *and in *GSVIVT0009456001 *gene models respectively. Moreover the gene models *GSVIVT00021812001*, *GSVIVP00013482001*, *GSVIVT00019482001*, and *GSVIVP00007524001 *were predicted to encode proteins that contained two or more ERF domains. Since both of these sequences were homologous to *Arabidopsis *proteins belonging to the ERF family and not to the AP2 family, we decided to split each gene model into sequences encoding a single ERF domain protein, using the closest Arabidopsis protein as a template (*GSVIVT00021812001: VvERF014 *and *VvERF016; GSVIVP00013482001: VvERF029 *and *VvERF030; GSVIVT00019482001: VvERF115, VvERF116 *and *VvERF119; GSVIVP00007524001: VvERF101 *and *VvERF102*). In order to test whether the prediction of these dubious genes was correct, we compared the production of amplicons corresponding to single exons with that of the predicted cDNA derived from exon-joining. The absence of an amplicon that corresponded to exon joining, together with the correct amplification of the single exon, was interpreted as an indication of misprediction. The gene model *GSVIVT00034010001*, which is supported by cDNA report evidence, was used as a positive control. No amplification was obtained using primers annealing to the N- and C- terminus of *GSVIVT00009519001*, *GSVIVT0009456001 *and *GSVIVT00021812001*, although an amplicon of the expected size was obtained using primers that anneal within the exons (Additional file 4, Figure S4). Since the present results revealed the possibility that other gene structure predictions were partly incorrect, all dubious sequences were checked and, where necessary, corrected, using *Arabidopsis *and poplar closest homologs as templates (Additional file 1, Table S1).

### Phylogenetic analysis

In order to investigate the evolutionary relatedness of the identified sequences, together with the ERF genes encoded by the other fully sequenced plant species, we performed a sequence-based phylogenetic analysis.

**Figure 1:**
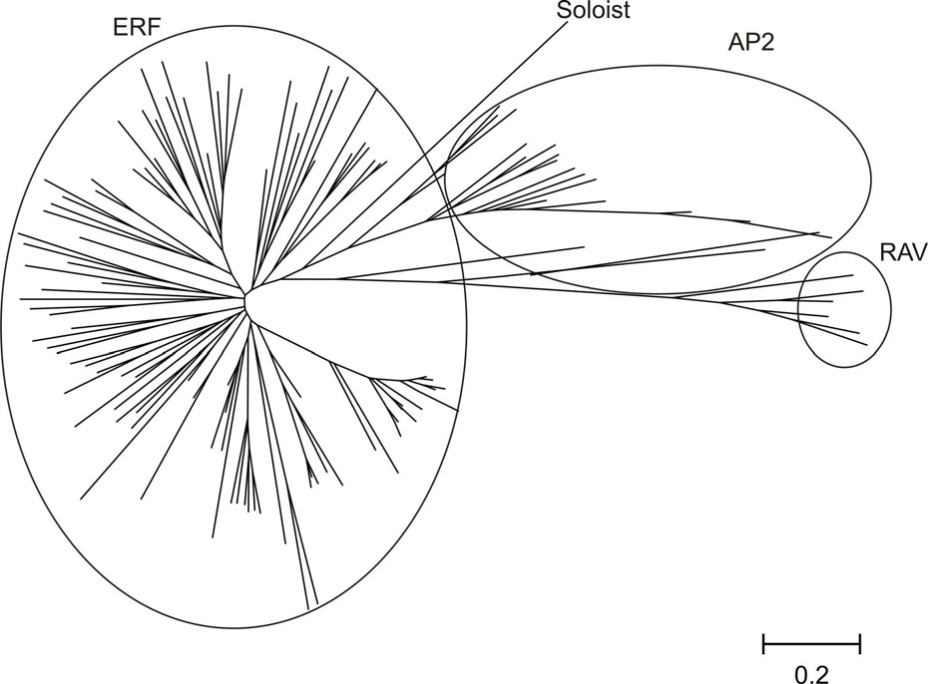
**The AP2/ERF superfamily in *Vitis vinifera***. Phylogenetic tree illustrating the relatedness of *Vitis vinifera *AP2/ERF proteins. An unrooted neighbour-joining tree was created using MEGA4 (Tamura *et al*., 2007). The distance bar is shown on the bottom of the tree.

The resulting phylogenetic tree (Figure 1) shows 15 clades, which correspond, according to Sakuma *et al. *[7] and Nakano *et al*. [8], to the ERF, the AP2 and the RAV superfamilies. The ERF superfamily is subdivided into 10 clades, which correspond to the group I-X as described by Nakano *et al*. [8]. Although the VvSoloist transcription factor contains a single AP2 domain, it clusters together with the AP2 superfamily. Zhuang *et al*. [23] identified two recent duplication events in the grapevine genome: one occurring for members of the B3 group (*VvERFB3-5 *and *VvERFB3-6*, corresponding to *VvERF099 *and *VvERF100 *in this study) and the second in the DREB group (*VvDREB-A4-12 *and *VvDREB-A4-13 *corresponding to *VvERF014 *and *VvERF016*, respectively). However, we did not find a perfect identity between *VvERF014 *and *VvERF016 *(Additional file 5, Figure S5). Instead, we identified 17 genes encoding for ERF-IX proteins characterized by a high sequence similarity (highlighted in red in Additional file 1, Table S1). Four of these genes (*VvERF080*, *VvERF082*, *VvERF083 *and *VvERF085*) maintained a high sequence similarity at a nucleotide level, outside the coding sequence approximately 50 downstream and 150 bp upstream (Additional file 6, Figure S6).

### Chromosome position of the identified *Vitis vinifera *AP2/ERF genes

**Figure 2:**
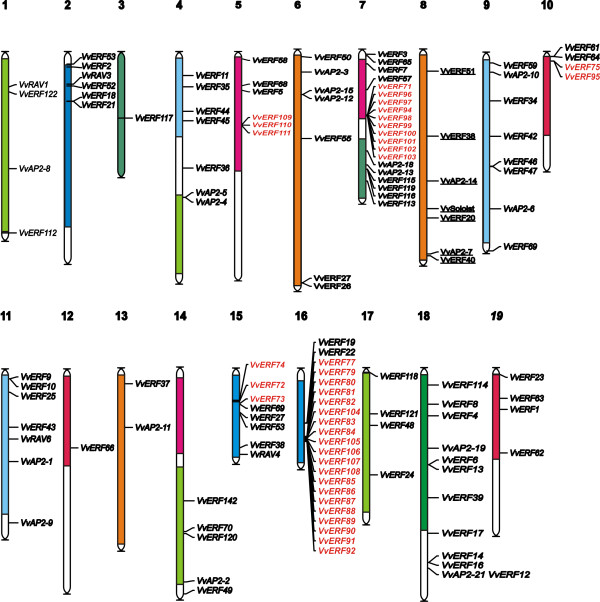
**Chromosomal locations of Vitis vinifera AP2/ERF MIKC genes**. Paralogous regions in the putative ancestral constituents of the *V. vinifera *genome are depicted in the same color as Jaillón *et al*. (2007).

The *AP2/ERF *genes were distributed unevenly among the nineteen chromosomes of the grapevine genome (Figure 2). Thirteen genes could not be assigned to any specific chromosome. Chromosomes 3 and 12 appeared to contain only one ERF gene each, followed by chromosome 13 (2 genes) and 1-10-17-19 (4 genes). The highest number of ERF genes was found on chromosomes 7 and 16, with 20 and 22 genes respectively. The high number of AP2/ERF sequences in these two chromosomes is mainly due to the presence of a close repetition of genes belonging to the same group. On chromosome 7, the ERF groups IX and X accounted for 25% and 40% of the total ERF genes, respectively. This feature is even more evident in chromosome 16, where group IX accounted for over 90% of the total amount of AP2/ERF genes. The repetition of genes in close distances seemed to be a typical feature of group IX. Interestingly, the same trend was observed for the chromosomal location of group IX (group B3 according to Sakuma *et al*., [7]) in the poplar genome [24]. As already observed for MIKC gene subfamilies [26], also AP2/ERF genes belonging to the same group were located in chromosomal regions, which have been suggested to represent paralogous segments resulting from ancestral polyploidization events [[20] and [22]]. For instance *VvERF018*, *VvERF019*, *VvERF022*, *VvERF027 *(group III) are located on chromosomes 2, 15, and 16; *VvAP2-1*, *VvAP2-6*, *VvAP2-9 *and *VvAP2-10 *are located on chromosomes 9 and 11. Closely related members of the group V (*VvERF042 *to *VvER0F47*) are located on the chromosomes belonging to the same paralogous segment. Moreover, 50% of the ERF group VIII is located on chromosomes 10, 12 and 19 (*VvERF061*, *VvERF062*, *VvERF063*, *VvERF064 *and *VvERF066*).

### Expression analyses of the AP2/ERF genes in aerial tissues of *V. vinifera *cv. *Corvina*

**Figure 3:**
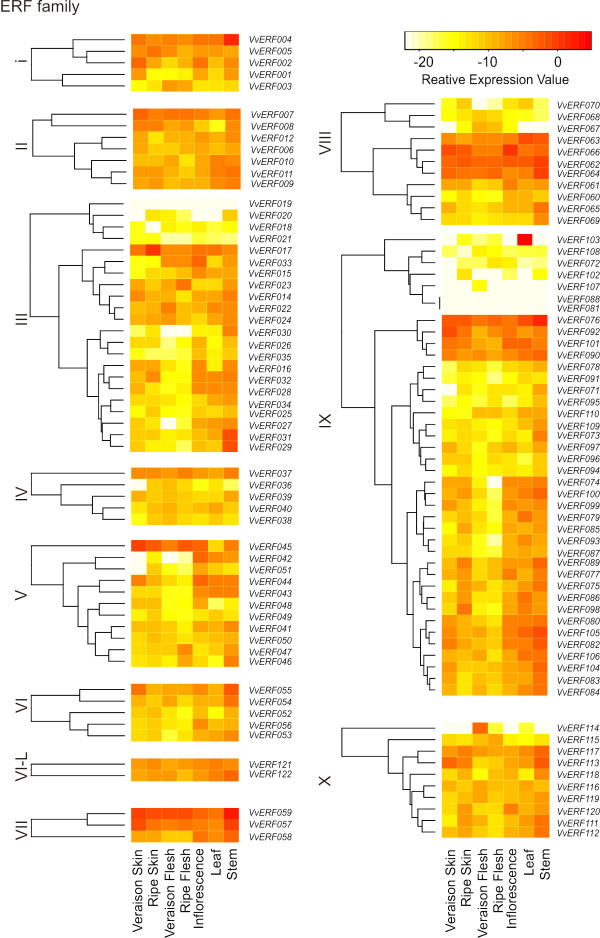
**Expression profile of *V. vinifera *ERF genes, subdivided into groups (I-X)**. The expression of all ERF genes identified in this study was measured by RT-qPCR in aerial vegetative (leaves and stems) and reproductive (inflorescence, berry skin and berry flesh) tissues. For berry and skin tissues two developmental stages (*veraison *and full ripeness) were analyzed. The relative expression value was calculated according to the formula Ct_HK_-Ct_Gene_. The results shown are from at least three independent replicates. Hierarchical clustering was used to represent the gene expression within each family.

**Figure 4:**
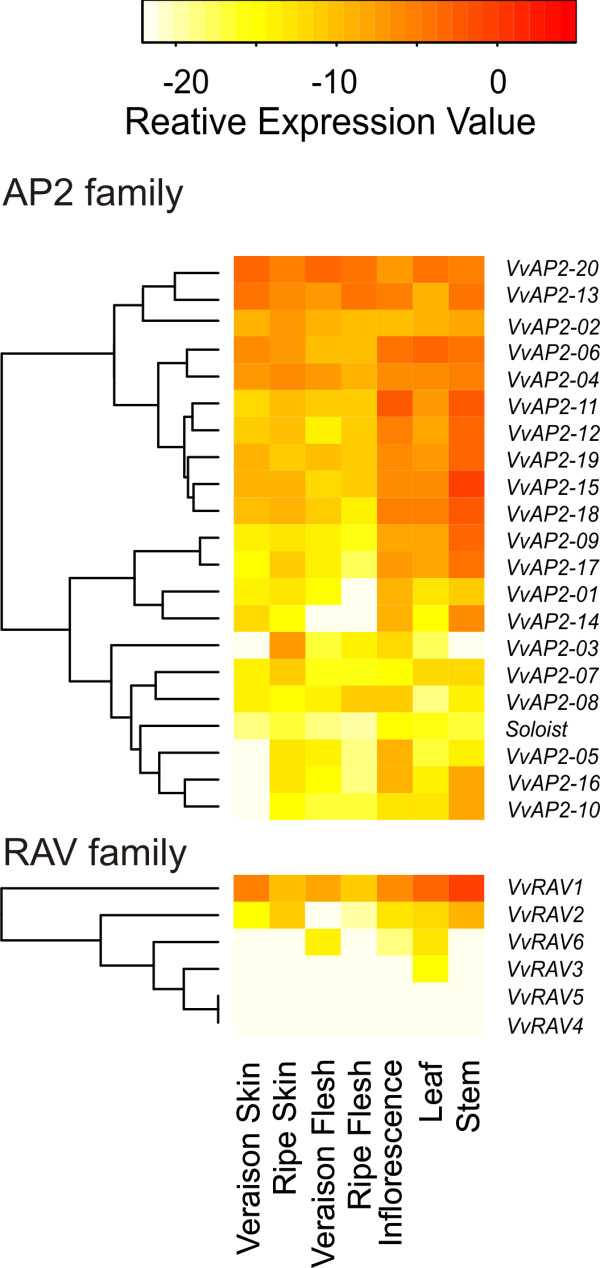
**Expression profile of *V. vinifera *AP2 and RAV genes**. The expresssion of all AP2 and RAV genes identified in this study was measured by RT-qPCR in aerial vegetative (leaves and stems) and reproductive (inflorescence, berry skin and berry flesh) tissues. For berry and skin tissues two developmental stages (*veraison *and full ripeness) were analyzed. The relative expression value was calculated according to the formula Ct_HK_-Ct_Gene_. The results shown are from at least three independent replicates.

Grape and wine production is strongly affected by environmental cues and pathogens during the development of the plant. Since *AP2/ERF *genes have been reported to play a role in plant development and in responses to biotic and abiotic stresses, they represent ideal candidates to investigate the molecular regulation of these processes. Although microarray platforms exist for the grape genome, realtime-qPCR (RT-qPCR) is a very sensitive and cost-effective technique to analyze the expression of genes with rather low expression levels, such as transcription factors [27]. We therefore designed a qRT-PCR platform to analyze the expression of all 149 *AP2/ERF *genes in the aerial vegetative and reproductive tissues of *Vitis vinifera *Cv. *Corvina *(Additional file 7, Table S7). The expression patterns of the genes encoding for members of the ERF, AP2 and RAV families are shown in Figure 3 (ERF family) and in Figure 4 (AP2 and RAV families). ERF family members (Figure 3) were subdivided according to their respective group (I to X). Only five genes (*VvERF019*, *VvERF081*, *VvERF088*, *VvRAV5 *and *VvRAV6*) did not reach a detectable expression level in any of the tissues considered. Fourteen genes (*VvERF004*, *VvERF005, VvERF007, VvERF037, VvERF057, VvERF059, VvERF062, VvERF063, VvERF064, VvERF076, VvERF117, VvERF121, VvERF122 *and *VvAP2-20*) showed high expression levels in all tissues analyzed, irrespectively of the developmental stage considered.

A broad variability of expression patterns within families and groups was observed: for instance, in the ERF group V, *VvERF042 *and *VvERF043 *were highly expressed in leaf, inflorescence and stem tissues while *VvERF046 *and *VvERF047 *reached high levels in the flesh of ripe berries (Figure 3). Very few genes exhibited a specific single tissue expression: *VvERF103 *mRNA was barely detectable except in leaf tissues, *VvERF114 *was highly expressed in flesh tissues of the berry at the *veraison *stage and *VvAP2-03 *showed a strong induction in skin tissues during ripening (Figure 4).

Three ERF genes displayed a high expression in leaves, stems and inflorescences and low levels in the berry tissues considered: *VvERF027 *(group III of the ERF family), *VvERF042 *and *VvERF043 *(ERF group V) (Figure 3), together with a big group of AP2 genes (*VvAP2-09, VvAP2-11, VvAP2-12, VvAP215, VvAP2-17, VvAP2-18 and VvAP2-19*) (Figure 4). Surprisingly, none of the AP2-coding genes, which have been reported to play a role in flower development, were exclusively expressed in the inflorescence tissues (Figure 4).

Expression pattern similarity did not mimic the sequence similarity of ERF group IX. For instance, *VvERF084 *and *VvERF085*, which share more than 95% similarity, exhibited different expression patterns in fruit tissues and *VvERF109 *displayed stem specificity whereas its closest paralog, *VvERF110*, was poorly expressed in the same tissue.

### Ripening regulated AP2/ERF genes in grape berry

AP2 and ERF related transcription factors are assumed to play a role in fruit development during the ripening process in several species [[28,29] and [30]]. To identify AP2/ERF genes whose expression changes during the transition from *veraison *to full ripeness (stage III according to Deluc *et al. *[31]), we analyzed the mRNA levels of 149 AP2/ERF genes identified in skin and flesh tissues.

**Figure 5:**
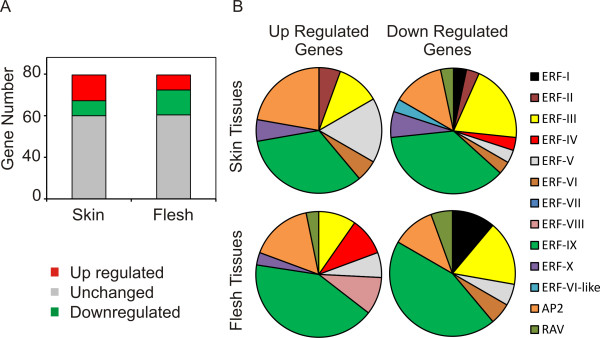
**Differentially regulated grapevine AP2/ERF genes during ripening**. (5A) Distribution of up-regulated, down-regulated and unchanged AP2/ERF genes in berry skin and flesh tissues. (5B) Distribution of up- and down-regulated AP2/ERF members in families and groups within the superfamily in skin and flesh tissues.

Three genes increased their mRNA levels during ripening in both *skin *and flesh berry tissues (*VvERF072*, *VvERF103 *and *VvAP2-3*) and only *VvERF018 *decreased its expression (Tables 2, 3). The highest number of differentially expressed AP2/ERF genes occurred in the skin tissues, where 31 genes were up-regulated at least 4 fold, and 18 genes were down regulated (≤-4 fold) (Table 2 and Figure 5). The opposite trend was observed in the flesh tissues, where 18 genes were induced and 30 repressed. In all groups of differentially expressed genes (DEGs), the ERF IX group was the most represented (Tables 2, 3), although only among the up-regulated ERF genes in the skin tissues the overrepresentation was significant (P-value≤0.05). Within the ERF family, at least one member from almost every group sequence changed its expression from *veraison *to full ripeness. In *Arabidopsis *and tomato (*Solanum lycopersicum*), transcription factors belonging to the ERF group IX, such as *AtERF1 *and *SlPti4*, are involved in the regulation of plant response to biotic and abiotic stresses mediated by ethylene and jasmonates [29,32]. Direct targets of AtERF1 include pathogenesis related proteins, chitinases and β-1,3-glucanases. Therefore it is tempting to speculate that the ERF genes belonging to group IX regulate proteins involved in stress responses in grapes.

**Table 2:** Differentially regulated AP2/ERF genes during ripening in the skin tissues.

Genes up-regulated in the transition from *veraison *to ripe in the skin tissues				Genes down-regulated in the transition from *veraison *to ripe in the skin tissues			
Name	Fold Change (log2)	**S.D**.	Family-Group	Name	Fold Change (log2)	**S.D**.	Family-Group
*VvAP2-3*	14.02		AP2	*VvERF001*	-8.6	2.62	ERF-I
*VvERF036*	11.17		ERF-IV	*VvERF018*	-5.87		ERF-III
*VvERF051*	10.04		ERF-V	*VvERF141*	-5.35	3.24	RAV
*VvAP2-5*	8.97		AP2	*VvERF002*	-4.86	2.25	ERF-I
*VvAP2-16*	8.71		AP2	*VvERF012*	-4.46	2.21	ERF-III
*VvERF94*	8.39		ERF-IX	*VvERF104*	-4.4	3.74	ERF-IX
*VvERF102*	8.34		ERF-IX	*VvERF055*	-4.35	3.48	ERF-VI
*VvERF098*	8.19	0.83	ERF-IX	*VvERF044*	-3.61	2.51	ERF-V
*VvERF020*	8.12		ERF-III	*VvERF082*	-3.6	2.14	ERF-IX
*VvERF042*	7.53		ERF-V	*VvERF092*	-3.55	0.92	ERF-IX
*VvERF111*	7.17	1.95	ERF-IX	*VvERF028*	-3.38	2.64	ERF-III
*VvAP2-10*	6.37		AP2	*VvERF084*	-3.29	1.16	ERF-IX
*VvERF067*	6.22		ERF-VIII	*VvAP2-14*	-3.15		AP2
*VvERF103*	5.91		ERF-IX	*VvERF108*	-2.82	1.36	ERF-IX
*VvERF077*	5.79	1.78	ERF-IX	*VvAP2-20*	-2.71	1.7	AP2
*VvERF093*	5.67	2.06	ERF-IX	*VvERF071*	-2.7	0.68	ERF-IX
*VvERF038*	5.28	1.58	ERF-IV	*VvERF105*	-2.45	1.72	ERF-IX
*VvERF097*	5.24	2.31	ERF-IX	*VvERF035*	-2.24		ERF-III
*VvERF017*	5.21	2.69	ERF-III				
*VvERF120*	5.18	0.59	ERF-X				
*VvERF070*	5.02	0.62	ERF-VIII				
*VvERF142*	4.58	0.55	RAV				
*VvAP2-17*	4.04	1.5	AP2				
*VvERF086*	3.93	1.68	ERF-IX				
*VvERF074*	3.59	1.94	ERF-IX				
*VvERF032*	3.59	0.7	ERF-III				
*VvERF040*	3.37	1	ERF-IV				
*VvERF072*	3.35		ERF-IX				
*VvERF060*	3.33		ERF-VIII				
*VvERF087*	2.08		ERF-IX				
*VvERF109*	2.03	0.39	ERF-IX				

Relative mRNA levels ERF, AP2 and RAV members up- or down-regulated in the skin tissues of the grapevine berry during transition from the *veraison *stage to full ripeness, quantified by RT-qPCR, according to the 2^-ΔΔCt ^method. The results shown are from at least three independent replicates. Expression values for which a standard deviation (SD) is not provided, correspond to samples that did not reach the selected threshold fluorescence level before the 39^th ^cycle in one of the two ripening stages.

**Table 3:** Differentially regulated AP2/ERF genes during ripening in the flesh tissues.

Genes up-regulated in the transition from *veraison *to ripe in the flesh tissues				Genes down-regulated in the transition from *veraison *to ripe in the flesh tissues			
Name	Fold Change (log2)	**S.D**.	Family-Group	Name	Fold Change (log2)	**S.D**.	Family-Group
*VvERF046*	6.46	0.65	ERF-V	*VvERF114*	-14.37		ERF-X
*VvERF118*	5.31	1.87	ERF-X	*VvERF146*	-7.94		RAV
*VvERF047*	5.2	0.64	ERF-V	*VvERF100*	-7.3		ERF-IX
*VvERF027*	4.69		ERF-III	*VvERF107*	-6.48		ERF-IX
*VvERF053*	4.5	3.5	ERF-VI	*VvAP2-1*	-6.07		AP2
*VvERF072*	4.27	1.61	ERF-IX	*VvERF099*	-5.85	5.29	ERF-IX
*VvERF071*	4.14	1.66	ERF-IX	*VvERF085*	-5.64	2.69	ERF-IX
*VvERF023*	4	0.81	ERF-III	*VvERF106*	-5.23	0.89	ERF-IX
*VvERF092*	3.45	1.88	ERF-IX	*VvAP2-5*	-4.9		AP2
*VvAP2-13*	3.35	1.35	AP2	*VvERF097*	-4.71		ERF-IX
*VvERF095*	3.13	1.09	ERF-IX	*VvERF010*	-4.64	2.29	ERF-II
*VvAP2-8*	3.13	0.39	AP2	*VvERF029*	-4.54	4.02	ERF-III
*VvAP2-3*	3.05		AP2	*VvERF022*	-4.44	1.46	ERF-III
*VvERF094*	2.81	1.81	ERF-IX	*VvERF111*	-3.96	0.03	ERF-IX
*VvERF103*	2.56		ERF-IX	*VvAP2-17*	-3.83	1.85	AP2
*VvERF045*	2.47	2.23	ERF-V	*VvAP2-16*	-3.53		AP2
*VvERF006*	2.45	1.35	ERF-II	*VvERF020*	-3.51		ERF-III
*VvAP2-12*	2.3	1.74	AP2	*VvERF054*	-3.49	0.08	ERF-VI
				*VvERF087*	-3.49		ERF-IX
				*VvERF079*	-3.25	2.53	ERF-IX
				*VvERF096*	-3.16	2.47	ERF-IX
				*VvERF016*	-3.13	2.01	ERF-III
				*VvERF05*	-3.12	2.4	ERF-I
				*VvERF122*	-3	1.09	ERF-6-L
				*VvERF117*	-2.81	0.6	ERF-X
				*VvERF024*	-2.42	1.07	ERF-III
				*VvERF040*	-2.38	0.41	ERF-IV
				*VvERF051*	-2.34	0.96	ERF-V
				*VvERF098*	-2.21	1.47	ERF-IX
				*VvERF018*	-2.09		ERF-III

Relative mRNA levels ERF, AP2 and RAV members up- or down-regulated in the flesh tissues of the grapevine berry during transition from the *veraison *stage to full ripeness, quantified by RT-qPCR, according to the 2^-ΔΔCt ^method. The results shown are from at least three independent replicates. Expression values for which a standard deviation (SD) is not provided, correspond to samples that did not reach the selected threshold fluorescence level before the 39^th ^cycle in one of the two ripening stages.

To test this hypothesis, we analyzed the expression of genes encoding for β-1,3 glucanases (*VvGlucβ-1*, *VvGlucβ-2*), class IV chitinase (*VvChit4-1*) and pathogenesis related proteins (*VvPR10.1 *and *VvPR10L-b*) using RT-qPCR. *VvGlucβ-1 *and *VvGlucβ-2*, *VvChit4-1*, *VvPR10.1 *and *VvPR10L-b *were induced in the transition from *veraison *to full ripeness in the skin of the berry (Additional file 8, Figure S8). The same trend was observed for several ERF-IX genes including *VvERF086*, *VvERF093 *and *VvERF098 *(Figure 3 and Additional file 8, Figure S8). Moreover, the expression profile of ERF-IX *VvERF078 *and *VvERF085 *is highly correlated with that of genes encoding chitinases, β 1,3-glucanases and thaumatin during different stages of berry development in the dataset published by Deluc *et al*. [31] (Additional file 9, Figure S9).

Only the VII group, which contains three members, was not represented in any of the DEG lists (Figure 3, Tables 2, 3). Four AP2 genes were induced and two were repressed in the skin during the ripening phase considered, whereas in the flesh only one gene was up-regulated and two were down-regulated (Tables 2, 3).

## Discussion

### Comparison of the AP2/ERF superfamily across sequenced plant genomes

In this study, a comprehensive search for genes encoding for AP2/ERF transcription factors encoded in the *Vitis vinifera *genome was carried out, leading to the identification of 149 genes. Previously, 132 AP2/ERF genes had been annotated, as reported by Zhuang *et al. *[23]. Eighteen newly identified genes encoded transcription factors belonging to groups II (*VvERF013*), III (*VvERF028 *and *VvERF029*), V (*VvERF041*), VIII (*VvERF060*, *VvERF061*, *VvERF062*, *VvERF063*, *VvERF071*, *VvERF073 *and *VvERF074*), IX (*VvERF094*) and X (*VvERF118 *and *VvERF121*) of the ERF family, to the AP2 family (*VvAP2-12 *and *VvAP2-17*) and the RAV family (*VvRAV3 *and *VvRAV5*) (Additional file 1, Table S1). Our analysis did not identify *GSVIVP00019513001 *as an ERF gene due to its short sequence and the presence of two long introns. It is likely that this locus constitutes a pseudogene. This number of genes is comparable with that of Arabidopsis but is only 70% of the number found in poplar and 80% of the rice AP2/ERF superfamily (Table 1).

The availability of the complete genome sequence of three dicot species and one monocot enabled a comparison of individual families and groups using Nakano *et al.*'s criteria [8]. Several ERF groups (II, III, VI, VII, VIII, IX, X, VI-L and Xb-L) and the AP2 family contained more members in poplar than in grape and *Arabidopsis *(Table 1). A feature common to both woody species *Vitis vinifera *and *Populus trichocarpa *is the lower number of genes in group I in the ERF family and the higher number in groups V and IX. In *Medicago truncatula*, *WXP1*, a group I ERF, contributes to drought tolerance by regulating the accumulation of cuticular waxes [33] while, in *Arabidopsis*, this function is performed by the SHYNE clade of ERF genes (*SHN1-3*), all belonging to group V [34]. Therefore, it is possible that the function of group I and V overlaps and the increase in group V in woody dicot species poises the lower number of group I genes. The expression of homologs of the *Arabidopsis *SHN clade, *VvERF043 *and *VvERF044*, in fruit skin tissues (Figure 3) is not unexpected, since waxy layers are produced to cover the grape berries [35].

Compared to all the species considered in this study, the grapevine genome has very small ERF-group II and VII. In *Arabidopsis*, the ERF-VII genes are highly expressed, suggesting to be regulated at the post-transcriptional level. Interestingly, also *VvERF057 *and *VvERF059 *are highly expressed in all the tissues analysed in this study.

ERF-IX is the largest ERF group in poplar and grape genomes, since it is almost twice as big as *Arabidopsis *and rice (Table 1). Of the 40 ERF-IX genes identified, 17 exhibited a high sequence similarity not limited to the coding sequence but also extended to the 5'and 3' surrounding regions (Additional file 6, Figure S6). However, since the expression profiles of these genes did not overlap completely, it is likely that their expression is driven by different promoter sequences. Interestingly, members of group IX displayed a particular clustered organization along chromosomes 7 and 16 (Figure 2), similar to the one observed in the poplar genome for the same group, although in this species the tandem repetition does not exceed four genes.

The tandem repetition of almost identical coding sequences suggests that these duplication events in the grapevine genome are quite recent. Moreover, it is tempting to speculate on the molecular mechanisms that led to the formation of such a long cluster of almost identical sequences, maybe involving the interaction of transposons or retroposons with the genomic sequence encoding ERF-IX genes. Magnani *et al*. [36] showed that a cyanobacterium (*Trichodesmium erythraeum*) possesses a HNH endonuclease protein with an AP2 DNA binding domain and suggested that the AP2 sequence approached the plant genome as a lateral transfer from a viral or bacterial donor. Gene duplication is more likely to be retained for gene families involved in signal transduction and transcriptional regulation [37]. Nakano *et al. *[8] suggested that an increased number of genes in groups III and IX might improve plant fitness since these genes are involved in the regulation of environmental stress responses. However, many other ERF groups, which do not have a high number of members, play a role in stress responses [8]. Therefore, the functional role of the ERF-IX group alone does not seem enough to explain the high number of genes in this family and their repetition on chromosomes. Further analyses of the nucleotidic and amino acid sequences of the genes belonging to this family are likely to shed light on this topic.

Genes belonging to the RAV family are highly conserved among dicot species, which contain six members, and not very different in rice (5 members) [38].

The number of AP2 genes is very similar between *Arabidopsis *(18) and *Vitis *(19) and lower than that of poplar (29 members) (Table 1). The generally higher number of AP2/ERF genes in the poplar genome is likely to be a consequence of a recent whole-genome duplication in the poplar lineage [39]. Curiously, a divergent member of the AP2 family, whose poplar homolog has been called Soloist, is maintained in a single copy in all plant genomes analyzed so far. The conservativeness of the sequence, plus the fact that it is only weakly related to other AP2/ERF genes, suggests that this gene diverged from the AP2 family early in the evolution of the plant species. Surprisingly, it also maintained its single copy feature during the separation of the monocot and dicot and the spread of the eurosid clade [20].

### Differential expression of AP2/ERF genes in aerial tissues of *Vitis vinifera*

The development of an RT-qPCR platform (Additional file 7, Table S7) encompassing the complete number of AP2/ERF genes previously identified, together with knowledge about the molecular function of their homolog in other plant species, may help in associating a role with some of these transcription factors.

Traditionally, the expression of ERF genes has been associated with the molecular response to ethylene, from which their acronym derives. Although AP2/ERF transcription factors are regulated by a number of physical-chemical stimuli, many ERF genes are indeed ethylene responsive and therefore have been suggested as being involved in the ripening process in climacteric fruits. Wine grape is considered to be a non-climacteric fruit but ethylene still plays an important role during development and ripening processes such as anthocyanin accumulation [40] and alcohol dehydrogenase expression [41].

Although many ERF genes have been shown to play a role in the response to abiotic and biotic stresses, only a few genes have been described as regulators of organ development. Two homologues of the development-related ERFs AtLEP and AtBOL, *VvERF067 *and *VvERF068*, are constantly expressed in the berry skin tissues, whereas a vitis homolog of the cytokinin responsive factors exhibits leaf-specific expression.

Many members of the groups II, III and IX (*VvERF085*, *VvERF087 *and *VvERF089*) were highly expressed in all the tissues examined (Figure 3). In Arabidopsis, tobacco (*Nicotiana tabacum*) and tomato (*Solanum lycopersicum*), ERFs belonging to groups II and III play a role in response to abiotic stress [[42] and [43]] whereas group IX includes genes involved in the response to pathogens [[44] and [45]]. The relatively high expression of members of these groups in grapevine tissues, under non stress conditions, may represent a default defence system, which was already present before the pathogen attack or the environmental stress took place, activated only when a trigger signal is perceived.

AP2 and RAV genes are the main actors in the determination of cell fate and development from meristems to fully developed organs. However, with the exception of *VvAP2-5*, the expression of AP2 and RAV genes in the tissues considered was not restricted to a single organ. On the other hand, *VvAP2-5 *is mainly expressed in the inflorescence tissues (Figure 4).

Contrary to what was expected, the expression homolog of the AP2 gene, *VvAP2-11, VvAP2-14 *and *VvAP2-15*, was not restricted to floral organs but extended to vegetative and fruit tissues (Figure 4). *VvAP2-9*, *VvAP2-11*, *VvAP2-12 *and *VvAP2-17*, which are homologs of the *Arabidopsis AIL-5*, *AP2*, *TOE1 *and *ANT *genes respectively, had a similar expression pattern, with the highest expression in the leaf, inflorescence and stem, but not in the fruit tissues (Figure 4). No member of the AP2 or RAV family showed a fruit-specific expression pattern.

Several ERF-IX members (*VvERF074*, *VvERF079*, *VvERF085*, *VvERF086*, *VvERF098*, *VvERF099*, *and VvERF100*) reached high mRNA levels in the skin tissues of the berry, but not in the flesh tissues (Figure 3). This differential expression between skin and flesh among members of group IX may reflect the need for a constantly activated defence in the skin of the berry, which is continuously exposed to pathogens attack. This was not the case however in the flesh tissues, where bacteria and fungi can only be present once the physical barrier of the skin has been penetrated.

### Expression changes of grapevine AP2/ERF genes in the *veraison *to full ripeness transition

Skin and flesh tissues from grape berries differ greatly during the transition from *veraison *to full ripeness with respect to ERF-gene expression. In the skin, 31 genes were up-regulated at least four-fold and 18 were down-regulated. In the flesh tissues, only 18 AP2/ERF genes were up-regulated and 30 down-regulated (Figure 5).

Group IX contained most of the genes induced or repressed by ripening in both skin and flesh tissues of the grapevine berry (Figure 5). Members of the same group are up-regulated in apple and plum fruit during ripening [28,46]. Deluc *et al*. [31] identified a small number of ERF IX as being differentially regulated throughout the development of the grape berry. This was either due to the sensitivity limitation of the microarray technique or because the transcriptome of skin and flesh tissues was not analyzed separately. The ripening-related induction of many members of group IX suggests that the skin cells need to be prepared against pathogen attack in a phase during which the berry is enriched with sugars and therefore represents the ideal substrate for pathogen growth. This hypothesis is supported by the fact that *VvGlucβ-1*, *VvGlucβ-2*, *VvChit4-1*, *VvPR10.1 *and *VvPR10L-b *were up-regulated during ripening, preferentially in the skin tissues, together with ERFs belonging to group IX (Additional file 8, Figure S8). In addition, in a more extensive series of developmental stages in whole berries (Deluc *et al*. [31]), the expression of stress-related genes correlated with that of ERF-IX members (Additional file 9, Figure S9). Moreover, increased levels of PR proteins and chitinases during the transition from *veraison *to ripening have been reported for grape *Barbera *cultivar [47]. In wine, PR proteins represent one of the most abundant classes of proteins [31]. Since they can negatively affect wine clarity and stability, their regulation may represent an important trait for improving wine quality.

The ERF-V *VvERF042 *and *VvERF046*, homologs of the Arabidopsis *SHN1 *gene, were induced in both skin and flesh tissues, while *VvERF044 *was repressed. An up-regulation of group V members during berry development was also reported by Deluc *et al*. [31]. In Arabidopsis, SHN1 regulates lipid biosynthetic pathways towards cutin synthesis [48], while a barley SHN1-homolog regulates lipid anabolism to generate organ adhesion [49]. Of the direct targets of SHN1, Kannangara *et al*. [48] identified the Long Acyl-coA synthetase 2 (*LACS2*), which is required for cutin biosynthesis in wild-type plants. Interestingly, in *Vitis vinifera *cv. Cabernet, *VvLACS7*, a homolog of the *Arabidopsis LACS2*, was reported to be up-regulated during ripening [48] and its expression highly correlates with *VvERF046 *in the same subset (Spearman correlation coefficient 0.92). An equal number of ERF-III genes was induced and repressed in the skin, whereas only two were up-regulated in the flesh tissues (Tables 2, 3). Group III encodes transcription factors involved in the crosstalk between biotic and abiotic stress [50] and therefore may play a similar role as group IX during the last phases of ripening in the berry. Members of group IV, which were also involved in stress response regulation, were induced in the skin but not in the flesh (Table 2). The ripening related induction of group VIII-ERF has already been reported in plums [[30] and [51]]. Also in the grape berry, three members were up-regulated in the skin tissues (Table 2).

Previous studies on ripening regulated transcripts identified members of the group VII as being associated with ripening [[28] and [46]]. However, in our transcriptional analysis, none of the three ERF-VII genes showed a differential expression in the berry skin or flesh in the transition from *veraison *to full ripeness (Figure 3). It is possible that the ripening-related induction of these genes occurs at earlier stages of the ripening process.

Two AP2 genes, *VvAP2-5 *and *VvAP2-16*, homologs to the Arabidopsis *PTL3 *(*AIL-6*) and *PTL4 *(*BBM*), respectively were strongly up-regulated in skin tissues during ripening, whereas an AIL-9 homolog, *VvAP2-9*, was repressed. In *Arabidopsis*, The PTL proteins act in a dose-dependent way to regulate stem cell maintenance and meristem boundaries in Arabidopsis, probably by PIN-dependent auxin distribution [[52] and [53]]. Their function in fruit development has not yet been studied; however they represent interesting candidates as regulators of the transition from different ripening stages. The *RAV *gene *VvRAV2 *was up-regulated in the skin tissues (Table 2). Its closest Arabidopsis homolog, *RAV2*, has been proposed to act as repressor, through the RLFGV motif located at the C-terminus [54]. Since VvRAV2 maintained the presence of this motif, it is possible to hypothesize a similar molecular activity.

## Conclusions

In summary, in this study we identified 149 AP2/ERF genes in the grape genome and characterized their expression patterns in aerial vegetative, inflorescence and berry skin and flesh at two different ripening stages, *veraison *and full ripening. A comparison of homologs from other species, whose genome has been sequenced, together with their expression profiles, may help in an understanding of the role of these transcription factors in perennial plants. *Vitis vinifera *represent the only woody plant whose genome is fully sequenced and whose commercial value is due to fruit production. Unveiling the role of AP2/ERF transcription factors in the developmental and ripening processes in this species, may help molecular breeders in improving fruit quality.

## Methods

### Identification of Vitis AP2/ERF genomic sequences

A search of the *Vitis vinifera *genome database was performed in order to find all members of the AP2/ERF family. A double strategy to obtain every gene of the AP2/ERF family in the genome was used. The sequences of all members of the ERF family in the genome of *Arabidopsis thaliana *were downloaded from the DATF database [55] and the amino acid sequence of one or most representative members (i.e. the maximum number of different conserved motifs distinctive of each group) for each group defined by Nakano *et al*. [8] were used as queries to search the grapevine genome database (Genoscope, CEA - Institut de génomique, France) using the BLAT program [56]. We also used the consensus sequence of the ERF domain to search the *Vitis *genome database on the NCBI web site. Every sequence identified was subsequently checked against the Arabidopsis and Poplar protein databases to confirm that it belonged to the AP2/ERF superfamily. As a final quality check, we confirmed the presence of the AP2 domain in every AP2/ERF *Vitis vinifera *gene candidate using SMART [57].

### Phylogenetic Analysis

Phylogenetic and molecular evolutionary analyses were conducted using MEGA version 4 [58]. To generate a phylogenetic tree, complete AP2/ERF predicted proteins *Vitis vinifera *were aligned using the ClustalW algorithm version 2.0 [59]. The neighbour-joining method was used to construct different trees, using the pair-wise deletion option. The reliability of the obtained trees was tested using bootstrapping with 1000 replicates.

### Gene prediction testing

Genomic sequences predicted to encode ERF genes that contained introns longer than 10 kb or that do not exist in Arabidopsis or poplar were considered as not reliable and four were (*GSVIVT00021812001*, GSVIVP00013482001, *GSVIVT00019482001*, and *GSVIVP00007524001*) chosen as examples. A primer pair annealing to the beginning and the end of the predicted coding sequence was used to check the reliability of the prediction. Primer pairs annealing within exons of the predicted coding sequence were used as controls to test that the gene considered was indeed expressed in the cDNA sample used. *GSVIVT00034010001 *was used as a positive control, since its coding sequence has also been predicted by EST analyses and its structure is strongly conserved by its homologues in different plant species such as potato, rice, Arabidopsis, sorrel (Licausi *et al. *unpublished).

### Gene Expression Analyses

Total RNA, extracted using the RNeasy kit (Qiagen) according to the manufacturer's instructions, was subjected to DNase treatment using the TURBO DNA-free kit (Ambion). Five micrograms of each sample were reverse transcribed into cDNA using the Superscript III reverse transcriptase kit (Invitrogen). Real-time PCR amplification was carried out with the ABI Prism 6900 sequence detection system (Applied Biosystems), using a power sybr-green master mix (Applied Biosystems) according to the manufacturer's instructions. Gene-specific primers (Additional file 7, Table S7) were designed on regions not interrupted by introns using Quantprime software [60], tested for the specificity of each amplification reaction. This was done by analyzing the dissociation curves of each amplicon and the primer efficiency was calculated using LinReg [61]. For all primer pairs and for all samples, the primer efficiency was between 1.7 and 2.0. Data were analyzed using 6900 SDS software 1.3 (Applied Biosystems). Dissociation curves for each amplicon were analyzed in order to verify the specificity of each amplification reaction. Transcript levels were normalized against the average of the grapevine housekeeping (HK) genes: *EF1- *gene (BQ799343), the ubiquitin gene (*VvUB*; CF406001), the actin gene (AB073011) and the glyceraldehydes dehydrogenase gene (EF192466). Relative gene expression in the transcriptomic analysis considering the different plant tissues was obtained by the formula Ct_HK_-Ct_Gene_, where Ct is the cycle number at which a reaction reaches a specified fluorescence level. Hierarchical clustering of the gene expression data was performed using R software. For the relative gene expression level related to the ripening, the ΔΔCt [62] method was employed. Statistical analysis of ERF group overrepresentation among differentially expressed genes was performed applying the Fisher's Exact test [63].

## List of abbreviations used

AIL-5: AIntegumenta Like 5; ANT: AINTEGUMENTA; AP2: APETALA 2; BBM: Baby Boom; BOL: Bolita; CBF: Cold responsive element binding factor; Chit4: class IV chitinase; DEG: Differentiallly Expressed Genes; DREB: Drought Responsive Elemenet Binding protein; DRN: DORNROSCHEN; DRNL: DORNROSCHEN-LIKE; ERF: Ethylene Responsive Factor; Glucβ: β 1-3, Glucanase; LACS: Long AcylCoA synthase; LEP: LEafy Petiole; PR10.1: Pathogen Related protein 10.1; PR10L-b: Pathogen Related protein b like; PTL: PeTal Loss; RAP2: Related To APETALA2; RAV: Related to ABI3/VP1; RT-qPCR: Real Time quantitative Polymerase Chain Reaction; SHN: SHYNE; TOE1: Target of EAT1 1; WXP1: WaXProduction1.

## Authors' contributions

FL and FMG carried out the *in silico *search of gene sequences in the grapevine genome. FL and SZ carried out expression analyses experiments from plant material to qRT-PCR. FMG performed the data analysis and statistics computation. FO participated in technical support. MP, FMG and PP participated in planning the experiments, writing the manuscript and revising. All authors read and approved the final manuscript.

## Supplementary Material

Additional file 1 **Complete list of ERF/AP2 genes identified in the *Vitis vinifera *genome**. Each ERF/AP2 sequence identified in this study is shown, together with the newly assigned name, the name assigned in Zhuang et al. (2009) [23], the best Arabidopsis hit, its family group (for ERF genes only), chromosomal location, CDS and predicted protein sequence. Click here for file

Additional file 2 **Phylogenetic tree of the "Soloist" homologues in grapevine, Arabidopsis and poplar**. Phylogenetic tree illustrating the relatedness of the aminoacidic sequences corresponding to the Soloist genes identified in grapevine (GIDVvP00018355001 in the present study and GSVIVP00025602001 according to Zhuang *et al*., 2009 [23]), Arabidopsis (At4g13040) and poplar (eugene3.00002518). Click here for file

Additional file 3 **List of common motifs present in the *Vitis vinifera *ERF genes**. List of common motifs (CM) identified by Nakano et al. (2006) [8] and present in the *Vitis vinifera *ERF genes. Click here for file

Additional file 4 **Accuracy of gene structure prediction for AP2/ERF genes in the Grapevine Genome Browser (X8)**. (1A) Gene structure of four *V. vinifera *ERF sequences taken as examples provided by the genoscope database. *GSVIVT00034010001*, whose structure is supported by cDNA sequencing (Grape Genome Browser [X8]), *GSVIVT000009519001*, *GSVIVT0009456001*, *GSVIVT00021812001*. Exons are depicted as empty squares and introns as single lines, with their size displayed at the top. Positions of the primers used for PCR amplification are indicated by arrows. Forward and reverse primers marked as "a" were used to test the presence of the complete sequence of the predicted cDNA. Primers marked with (b) were used in combination with the corresponding forward or reverse "a" primers to test the presence of the same cDNA. (1B) PCR-products obtained using the "a" and "a+b" primers with stem tissue cDNA as template. Click here for file

Additional file 5 **Pairwise alignment of the aminoacidic sequence of VvERF014 and VvERF016**. Pairwise alignment of the protein sequences corresponding to *VvERF014 *and *VvERF016*. Click here for file

Additional file 6 **Alignment of the genomic sequence of four homologous genes coding for ERF-IX TFs**. The initial ATG codon and the terminal stop codon TGA are shown in red. Click here for file

Additional file 7 **List of primers used in the present study**. Sequences of the oligonucleotides used as primers for the mRNA quantification and to test the gene structure prediction Click here for file

Additional file 8 **Expression of putative targets of ERF-IX transcription factors during ripening**. Relative mRNA levels of genes putatively involved as targets of ERF-IX members were measured by qRT-PCR in skin and flesh tissues of *Vitis vinifera *(cv. Corvina) berry at the veraison and full ripeness stage. *VvGlcβ-1*: β 1,3 glucanase 1; *VvGlcβ-2*: β 1,3 glucanase 2; *VvPR10L-b*: Pathogenesis Related protein 10-like B, *VvPR10.1*: pathogenesis related prtein 10.1; *VvChit4-1*: Chitinase 1-4. The results shown are from at least three independent biological replicates. Click here for file

Additional file 9 **Correlation of the expression of group-IX ERFs and their putative target genes**. Correlation of the expression of group IX-ERFs and their putative target genes. *1,4-D-glucanase *(CF212592), *CHIV1 *(AY137377), *1,3-Glucanase3 *(TC62849), *1,3-Glucanase1 *(CF605842), *thaumatin-like3 *(TC56535) and *CHIV2 *(TC64563). The Pearson Correlation coefficient (PC) was calculated using the expression values provided by Deluc *et al. *(2007) [31]. Click here for file
