# Supplementary material for: Genomic and transcriptomic analysis of the AP2/ERF superfamily in Vitis vinifera
Source: BMC Genomics. 2010 Dec 20;11:719. doi: 10.1186/1471-2164-11-719 (PMC3022922; doi:10.1186/1471-2164-11-719)

Additional Figure S2. Phylogenetic tree representing the relatedness of the aminoacidic sequences corresponding to the Soloist genes identified in grapevine (GIDVvP00018355001 in the present study and GSVIVP00025602001 according to Zhuang *et al*., 2009), Arabidopsis (At4g13040) and poplar (eugene3.00002518).

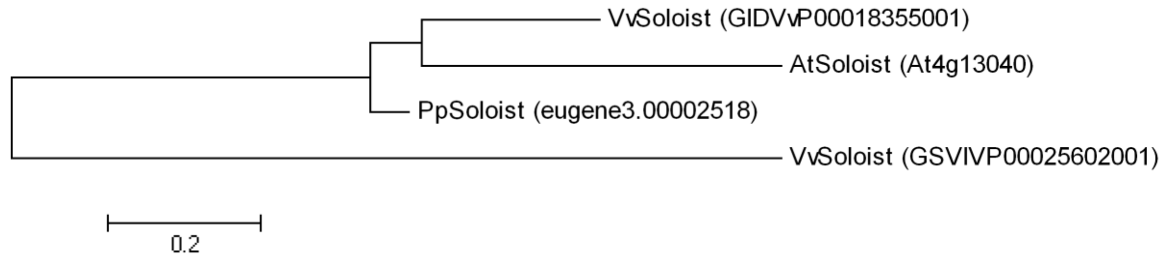

Supplement: Additional file 2 — Phylogenetic tree of the "Soloist" homologues in grapevine, Arabidopsis and poplar. Phylogenetic tree illustrating the relatedness of the aminoacidic sequences corresponding to the Soloist genes identified in grapevine (GIDVvP00018355001 in the present study and GSVIVP00025602001 according to Zhuang et al., 2009 [23]), Arabidopsis (At4g13040) and poplar (eugene3.00002518). [file 1471-2164-11-719-S2.PDF]
