# Supplementary figures and images for: Genomic and transcriptomic analysis of the AP2/ERF superfamily in Vitis vinifera
Source: BMC Genomics. 2010 Dec 20;11:719. doi: 10.1186/1471-2164-11-719 (PMC3022922; doi:10.1186/1471-2164-11-719)

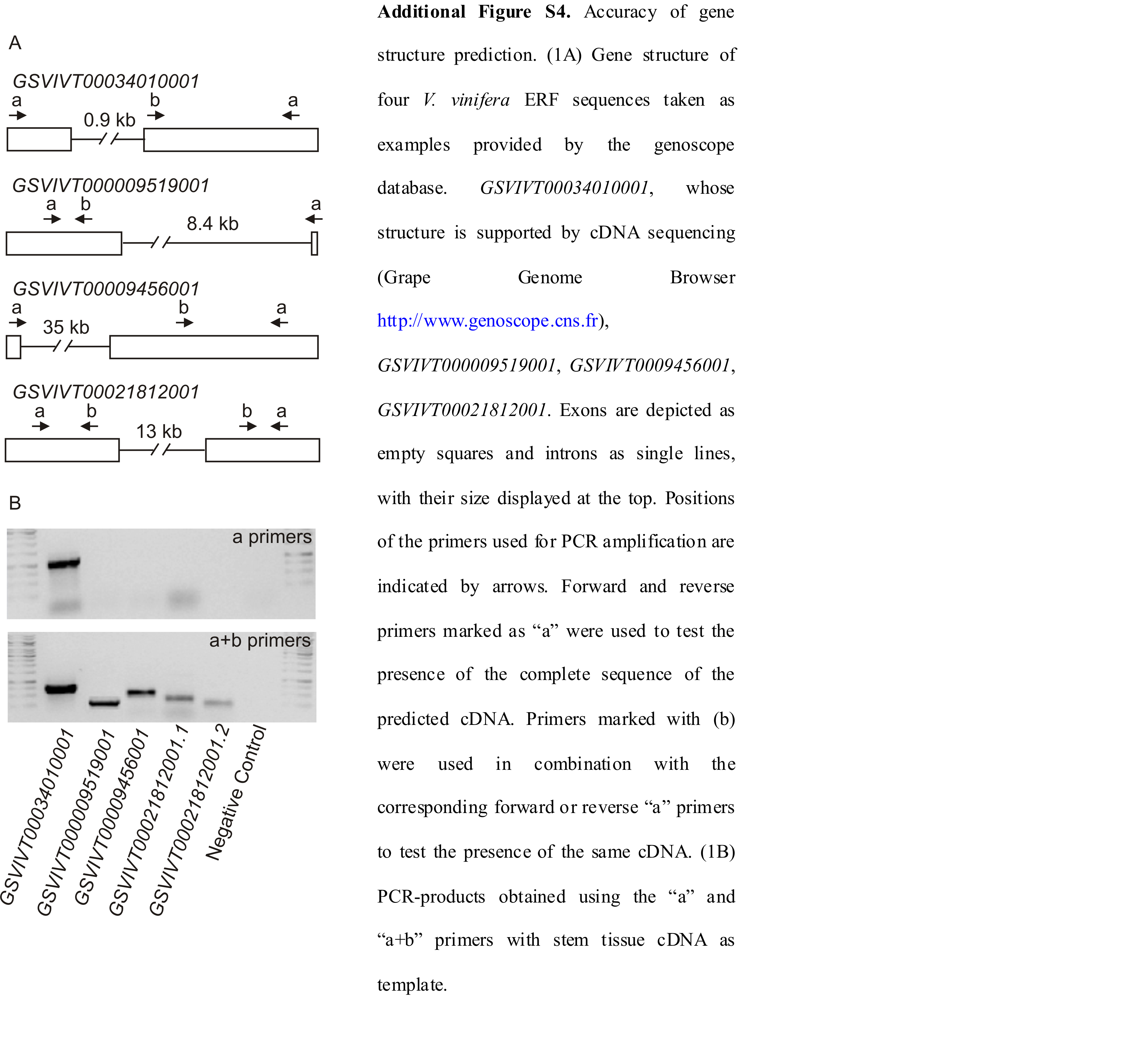

Supplement: Additional file 4 — Accuracy of gene structure prediction for AP2/ERF genes in the Grapevine Genome Browser (X8). (1A) Gene structure of four V. vinifera ERF sequences taken as examples provided by the genoscope database. GSVIVT00034010001, whose structure is supported by cDNA sequencing (Grape Genome Browser [X8]), GSVIVT000009519001, GSVIVT0009456001, GSVIVT00021812001. Exons are depicted as empty squares and introns as single lines, with their size displayed at the top. Positions of the primers used for PCR amplification are indicated by arrows. Forward and reverse primers marked as "a" were used to test the presence of the complete sequence of the predicted cDNA. Primers marked with (b) were used in combination with the corresponding forward or reverse "a" primers to test the presence of the same cDNA. (1B) PCR-products obtained using the "a" and "a+b" primers with stem tissue cDNA as template. [file 1471-2164-11-719-S4.TIFF]

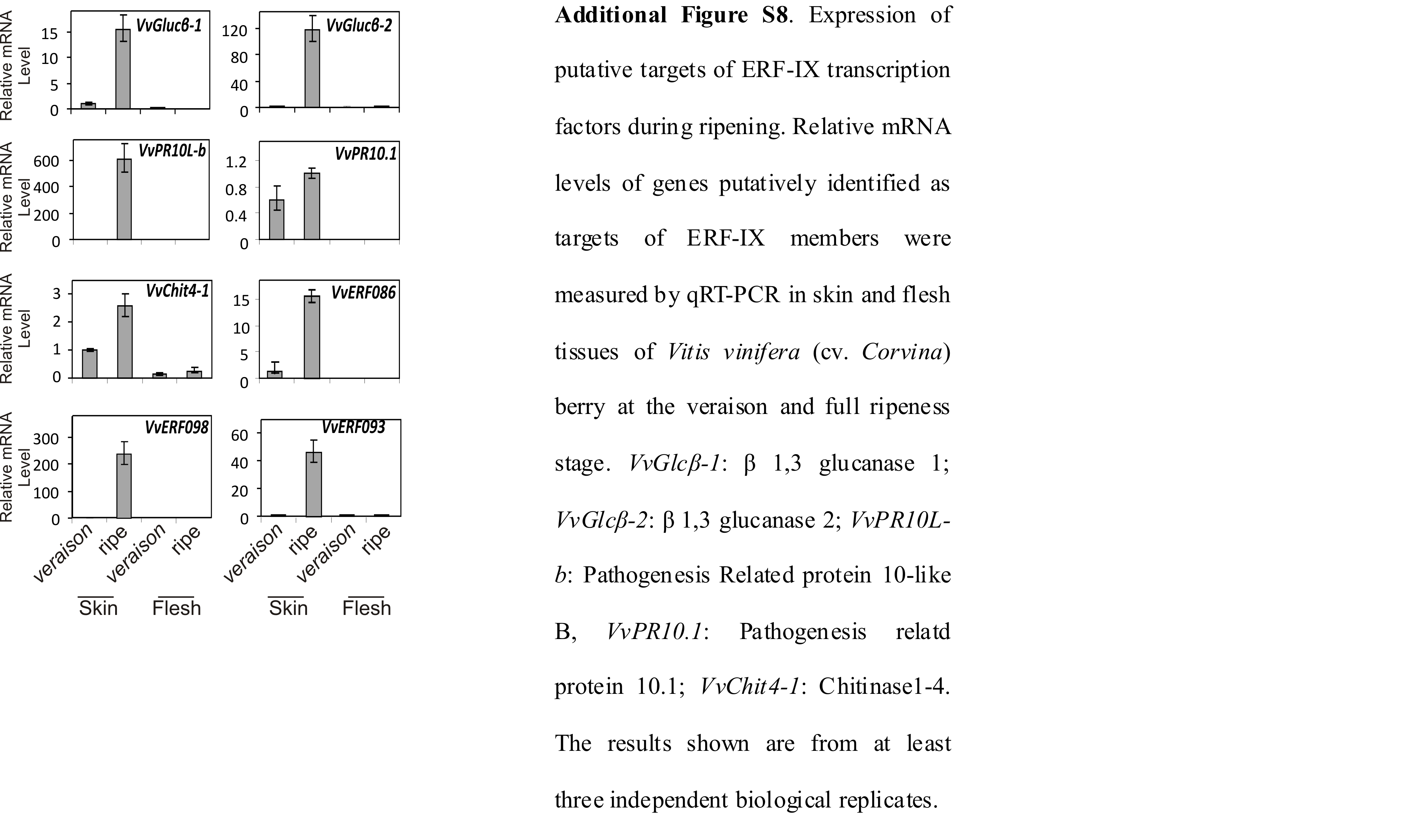

Supplement: Additional file 8 — Expression of putative targets of ERF-IX transcription factors during ripening. Relative mRNA levels of genes putatively involved as targets of ERF-IX members were measured by qRT-PCR in skin and flesh tissues of Vitis vinifera (cv. Corvina) berry at the veraison and full ripeness stage. VvGlcβ-1: β 1,3 glucanase 1; VvGlcβ-2: β 1,3 glucanase 2; VvPR10L-b: Pathogenesis Related protein 10-like B, VvPR10.1: pathogenesis related prtein 10.1; VvChit4-1: Chitinase 1-4. The results shown are from at least three independent biological replicates. [file 1471-2164-11-719-S8.TIFF]
