# Supplementary material for: Genomic and transcriptomic analysis of the AP2/ERF superfamily in Vitis vinifera
Source: BMC Genomics. 2010 Dec 20;11:719. doi: 10.1186/1471-2164-11-719 (PMC3022922; doi:10.1186/1471-2164-11-719)
Supplement: Additional file 5 — Pairwise alignment of the aminoacidic sequence of VvERF014 and VvERF016. Pairwise alignment of the protein sequences corresponding to VvERF014 and VvERF016. [file 1471-2164-11-719-S5.PDF]

Additional Figure S5. Pairwise alignment of the aminoacidic sequence of VvERF014 and VvERF016

```
VvERF014        -----MKNRADPHAPIPSYRGVRKRKWGKWVSEIREPGKKTRIWLGSYEAPEMAAAAYDV
VvERF016        MEDDQGHSHGGAVASSSGYRGVRKRRWGKWVSEIREPGTKTRIWLGSFETPEMAAMAYDA
                      :.:... *. ..*******:************.********:*:***** ***.

VvERF014        AALHLKGHRAELNFPELAPSFPRPTSFSPEDVQFASAQAALHIKSIASGDVSARSPSRVG
VvERF016        AALHFRGHGAKLNFPELAPNLPRPASSAAEHIRLAAQEAALRLTRAPESAGQSGS----G
                ****::** *:********.:***:* :.*.:::*: :***::.  ...  .: *    *

VvERF014        MSSVPTRVGLSASEIREINEWPMDSPRMWMMELR---EEPLVLSDEIFELSEWEEVENQY
VvERF016        SGLGPVRVGLSPSQIQAINDSTLDSPRNWTELAQTVFLDESMIMSCELEISDWDEEEVEV
                 .  *.*****.*:*: **: .:**** *    :    :  :: .  :*:*:*:* * :

VvERF014        SIWDL
VvERF016        N----
                .
```
