# Supplementary material for: Genomic and transcriptomic analysis of the AP2/ERF superfamily in Vitis vinifera
Source: BMC Genomics. 2010 Dec 20;11:719. doi: 10.1186/1471-2164-11-719 (PMC3022922; doi:10.1186/1471-2164-11-719)
Supplement: Additional file 6 — Alignment of the genomic sequence of four homologous genes coding for ERF-IX TFs. The initial ATG codon and the terminal stop codon TGA are shown in red. [file 1471-2164-11-719-S6.PDF]

**Additional Figure S6.** Alignment of the genomic sequence of four homologous genes coding for ERF-IX TFs. The initial ATG codon and the terminal stop codon TGA are shown in red.

```
VvERF83         AAAATTGCTTTAAATTTTTTTAAAATTGATATTGTAAAATTTTTTAATATCTCAAATTT 60
VvERF85         ---------------------------CGATTTTGTAATTTTTTTT----TTCTAAAGAG 29
VvERF80         ---TTCATAGCCTTCCCCTCTTAGTTACAATTATGCAATTATCCTA----TTTATTGTTA 53
VvERF82         ---------GCCTTCCCCTCTTAGTTACAATTATGTAATTATCCTA----TTTATTGTTA 47
                                             **  ** **   *  *     *

VvERF83         GGTATAATAAAAAATTTTCATATCTCAAGTTTTTATTGTT--TTATTTTTTTATTTAAAA 118
VvERF85         CACCTCAAATTAAGATATAATTTGTTAACCCCTTGATGGTGATTGTTTTCTTATTTAATA 89
VvERF80         TTTTTTCTGCAGAATTTTAGCTTTAAAATGTTATAAAGTTG--AATTAAATAAATAAAAA 111
VvERF82         TTTTTTCTGCAGAATTTTAGCTTTAAAATGTTATAAAGTTG--AATTAAATAAATAAAAA 105
                    *       *  * *    *   **     *   * *     **   * * * ** *

VvERF83         AAAATCAGTAACTTTTCAATATAAATCTTTAAGATTACTTGCATTTTATA-AATAATGTT 177
VvERF85         TTAAGTA-TAATTTGTT--TTCAAATCTT---AATTAGTTGTTTTCTA-A-AGTTTTTTT 141
VvERF80         ATAGTTATTCATTGGGCGGTAGCAACTGGGCACGCCAGCTGGTGGGCATGTGGTTGAGGG 171
VvERF82         ATAGTTATTCATTGGGCGGTAGCAACTGGGCATGCCGGCTGGTGGGCATGTGGTTGAGGG 165
                  *   * * * *      *   **              **      *     *

VvERF83         ATTAATTTTA--AAATATATATATATATATAATTTGTTCTCACTTGGGTGGGTTCAATTA 235
VvERF85         CTTAATTTTT--AATT-TATATATATATATATATATATGACTTTTCT---CTTAATCT 195
VvERF80         GGAAACTATGCGGATGGAACCCATAGGAGAGACCCAAGTTGAGAAAGCTGT-CCGGTCCA 230
VvERF82         GGAAACTATGCGGATGGAACCCATAGGAGTGACCCAAGTTGGGAAAGCTGT-CCGGTCCA 224
                   ** * *    *    *   ***              *        *

VvERF83         AGGGTGAGGATTACGATGATGC-GTCATTA---TAATCGATCCAAACCCCAAAGCGTATA 291
VvERF85         AAAAAGTAATTTAATTTAACATTAACTTAA---AAAACCAAACATCACCTTAATTTCAAC 252
VvERF80         AGGGGATAAACCAGCTTCATCCAGAGTCAGATCTTATCATTGAATAGTATTGG------- 283
VvERF82         AGTGGATAAACCAGCTTCATCCAGAGTCAGATCTTATCATTGAATAGTATTGGTCCTATT 284
                *           *   * *                *  *     *

VvERF83         TAGC-CTTTTCTATT--GGTTGTGGTCCCTTTTGCATTCTAAAG-AAAGCACCCTCTGGT 347
VvERF85         TAAC-CGTGAAAATTAGGATGGTGGGTCATTATAATCCATACCCCAAAGCATATATAGCC 311
VvERF80         --ACACAAAATAAGTCAAAATATAGGATCTCAGAGTCTCTGTATTAAACCATAAGTCCCT 341
VvERF82         GGACACAAAATAAGTCAAAATATAGGACCTCAGAGTCTCTGTATTAAACCATAAGTCCCT 344
                   * *      * *       * *    *         *     *** **

VvERF83         CGTCTGTTTTGTTGGAGGTAAAAAAAAAATACATATACATGCATGGTCCCT-CCACAGATT 406
VvERF85         TTTTCTATTTGTTGTGGTCCCTTTTGCATACATAGACATGCATGGTCCCT-CCACAGATT 370
VvERF80         CTATACATGTGGGAAGAACCCAAGAAACCACTGTCGGACTAATAATATAAACCATAAGTT 401
VvERF82         CTATACATGTGGGAAGAACCCAAGAAACCACTGTCGGACTAATAATATAAACCATAAGTT 404
                       * **                  **      *   **  *     *** *  **

VvERF83         AAAGAACGTATATTCAAACCCCACTTTTTTCA-ATGGA-------AAGAAGCTCCATTCC 458
VvERF85         AAAGAATGTATATACAAACCCCACTTTTTTCA-ATGGA-------AAGAAGCTCCATTCC 422
VvERF80         TGAATATATAAATCCCACTTGCGATGTTGACATATGGATACCACAAAGAAGTTTCATTCC 461
VvERF82         TGAATATATAAATCCCACTTGCGATGTTGACATATGGATACCACAAAGAAGTTTCATTCC 464
                  *  *  ** ** * *    *  * **  ** *****       ****** * ******

VvERF83         CCCAACCAGGAACCTCGTCCGAAACCTCCTCTCTCTATCTAAGATGTTACGCACAACTCT 518
VvERF85         CCCAACCAGGAACCTCATCCGAAACCTCCTCTCTCTATCTAAGATGCAACGCATAACTCT 482
VvERF80         CCCGACTTGGAACCTCATCTGAAGCCTCCTCTTTCTATCTATCGTGTTATATATAGCTCC 521
VvERF82         CCCGACTTGGAACCTCATCTGAAGCCTCCTCTTTCTATCTATCGTGTTATATATAGCTCC 524
                *** **  ******** ** *** ******** ********   **  *   * * ***

VvERF83         GTTATATATAGCTCCACCACAC--------------------ACTCTCTTTGCTTGTTTA 558
VvERF85         GTTATATATACCTCCACCACACTCTCCTTCCCACTCACACTCACTCTCTTTGCTTGTTTA 542
VvERF80         ACCACAC-TCTCTCCTCTCCACTC------------------ACTCTCACTCTCTACTT- 561
VvERF82         ACCACAC-TCTCTCCTCTCCACTT------------------ACTCTCACTCTCTACTT- 564
                   * *  *  **** *  ***                    ******  *   *  **

VvERF83         ATGTTTTAATACTTCACTATTTCTA--------------TTCATATGGCAGAAGAAGCTT 604
VvERF85         GTGTTTTAATACTTCACTATTTCTAAGTGCTCATCTCCATTAATATGGCAGAAGAAGCTT 602
VvERF80         ----CTCAGTGCTC----ATCTCCA--------------TTGATATGGCAGAAGAGGCTT 599
VvERF82         ----CTCAGTGCTC----ATCTCCA--------------TTGATATGGCAGAAGAAGTTT 602
                     * * * **    ** ** *              ** ************* * **
```

```
VvERF83         CATCACTGCACCTCATCCACCAACTACTCCTCTCCGACTTTCAATCTTTCGAAAATTT-- 662
VvERF85         CATCACTGCACCTCATACACCAACTACTCCTCTCCGACTTTGAATCTTTCGAAAATTT-- 660
VvERF80         CATCGGTTCACTTCATTCACCAACAACTTCTCTTCGACTTTGAAGCTTTCGAAAGCTTCG 659
VvERF82         CATCGGTTCACTTCATTCACCAACAACTTCTCTTCGACTTTGAAGCTTTCGAAAGCTTCG 662
                ****  * *** **** ******* *** **** ******* ** *********  **

VvERF83         ----------AAATGATCCATCCCAAACTTCAACCTCTGATTCGAGTATTTCCACCTCAG 712
VvERF85         ----------AAATGATCCATCCCAAACTTCAACCTCTGATTCCAGTGTTTCCACCTCAG 710
VvERF80         TCTCTCATGTAAATGATCCATCCCAAACTTCAACCTCTGATTCGAGTGCTTCCACCTCAG 719
VvERF82         TCTCTCATGTAAATGATCCATCCCAAACTTCAACCTCTGATTCGAATGCTTCCACCTCAG 722
                          ********************************* * *  ***********

VvERF83         ATATTATTCCGCTCTCTAATTACTTCGATCTCCATGAAGATGAAAACAACCCCTTTCTCC 772
VvERF85         ATATTATTCCGCTCTCTAATTACTTCGATCTCCATGAAGATGAAAACAACCCCTTTCTCC 770
VvERF80         ATATTAATCCGCTCTCTAATTACTTCAATCCCCATGAAGATGAAAACAACCCCTCTCTCC 779
VvERF82         ATATTAATCCGCTCTCTAATTACTTCAACCCCCATGAAGATGAAAACAACCCCTCTCTCC 782
                ****** ******************* * * *********************** *****

VvERF83         TCCACTGTTCTACTTCTGCTCCGTCTGGGTTTTTCCAGTTTGAATCCAAATCCCCCAAAG 832
VvERF85         TCCACTGTTCTACTTCTGCTCCATCTGGGTTTTTCCAGTTTGAATCCAAATCCCCAAAAG 830
VvERF80         TCCACTGTTCTACCTCTGCTCCGTCTGGCTTTTTCCAGCTTCAAACCAAATCCCCCAAAA 839
VvERF82         TCCACTGTTCTACCTCTGCTCCGTCTGGCTTTTTCCAGTTTCAAACCAAATCCCCCAAAA 842
                ************* ******** ***** ********* ** ** ********** ***

VvERF83         CTTCCAGATTAAGTCGTCGGCGACCTCCACTCAGCATCTCAGTTTCTCAGCCAACCATTC 892
VvERF85         CTTCCAGATTAAGTCATCGACGACCTCCACTCAGCATCTCAGTCCCTCTTCCCACCGTTT 890
VvERF80         CTTCCACATTAAGTCATCGGCGACCTCCACTCAGCATCTCAGTCCCTCAGCCCACCGTTT 899
VvERF82         CTTCCACATTAAGTCATCGGCGACCTCCACTCAGCATCTCAGTCCCTCAGCCCACCTTTT 902
                ****** ******** *** ***********************  ***  ** *** **

VvERF83         CTCAGTTGCCGGCGGATTCCGATTCAGGCGACGTCAAGCATTATAGGGGGGTACGGCGTC 952
VvERF85         CTCAGTCGCAGGGGGAGTCCGATTCAGGAAATATCAGGCTTTATAGAGGCGTGCGCCGAC 950
VvERF80         CTCAGTCACCGGCGGAGTCCGATTCAGGCGATATCAGGCATTACAGAGGCGTGAGGAGAC 959
VvERF82         CTCAGTCACCGGCGGAGTCCGATTCAGGCGATATCAGGCATTACAGAGGCGTGAGGAGAC 962
                ******  * ** *** ***********  *  *** ** *** ** ** **  *  * *

VvERF83         GGCCGTGGGGGAAATTTGCGGCGGAGATCCGAGACGCGAATCGGAGAGGATCGAGGGTAT 1012
VvERF85         GGCCATGGGGGAAATTCGCGGCGGAGATTCGAGACCCGAATCGGAGAGGATCGAGGGTAT 1010
VvERF80         GGCCATGGGGGAAATTTGCGGCGGAGATTCGAGACCCGAATCGGAGAGGATCGAGGGTAT 1019
VvERF82         GACCATGGGGGAAATTTGCGGCGGAGATTCGAGACCCGAATCGGAGAGGATCGAGGGTAT 1022
                * ** *********** *********** ****** ************************

VVERF83         GGCTGGGGACATTCGAGACTGCCATTGAAGCCGCCAGAGCTTATGATCGAGCTGCTTTCG 1072
VVERF85         GGCTGGGGACATTCGAGGCTGCCATTGAAGCCGCCAGAGCTTATGATCGAGCTGCTTTCA 1070
VVERF80         GGCTGGGGACATTCGAGACTGCCATTGAAGCCGCCAGAGCTTATGATCGAGCTGCTTTTG 1079
VVERF82         GGCTGGGGACATTCGAGACTGCCATTGAAGCCGCCAGAGCTTATGATCGAGCTGCTTTTG 1082
                ***************** ****************************************

VvERF83         AGATGCGTGGTTCCAAAGCTATTCTCAATTTCCCTCTTGAAGCTGGGAATTGGTCGGGTT 1132
VvERF85         AGATGCGTGGTTCCAAAGCTATTCTCAATTTCCCTCTTGAAGCTGGGAATTGGTCGGGTT 1130
VvERF80         AGATGCGTGGCTCCAAAGCTATTCTCAATTTCCCCCTTGAAGCTGATAATTGGTCGGGAT 1139
VvERF82         AGATGCGTGGCTCCAAAGCTATTCTCAATTTCCCCCTTGAAGCTGATAATTGGTCGGGAT 1142
                ********** *********************** **********  *********** *

VvERF83         CTGAATCACCAGTAATATCAGGCCAGAAAAGGGTGAGAGAAAGTGAGGGTGAAGAGAGAG 1192
VvERF85         CTGACCCACCAGCGATATCTGGCCAGAAAAGGGAGAGAGAGCGTGAGAGTGAAGGGAGAG 1190
VvERF80         CTGATCCACCAGCGATATCTGGCCGGAAAAGGGAGAGAGACAGTGAGACTGAAGAGAGAC 1199
VvERF82         CTGATCCACCAGCGATATCTGGCCGGAAAAGGGAGAGAGACAGTGAGACTGAAGAGAGAC 1202
                ****  ******  ***** **** ******** ******  *****  ***** ****

VvERF83         AACAAGTGGAAATTAAGGTTTTGAAGCAGGAGGAGCAGTCGCCGGAATCTGAGAGCACGG 1252
VvERF85         AACTAGTGGAAAATAAGGTTTTAAAGAAAGACGAGCAGTCTTCGGAATCTGAGAGCACGG 1250
VvERF80         AACAAGTGGAGATTAAGGTTTTAAAGCAAGAGGAGTACTTGCCCGAATCCGACAGCACGC 1259
VvERF82         AACAAGTGGAGATTAAGGTTTTAAAGCAAGAGGAGTACTTGCCCGAATCCGACAGCACGC 1262
                *** ****** * ********* *** * ** *** * *   * ***** ** ******
```

```
VvERF83      TGGCGGGG---GCAACGAGTAATGTTTTGGGGATTGGTCCGTTAACTCCGTCAAATTGGA 1309
VvERF85      TGGCGGCGGCGGCAGCCAGTAATTCTTTAGGGGTTAGTCCACTAACCCCTTCAAATTGGA 1310
VvERF80      TGGCGG------CAGCCAGTCATGTTTTAGGGGTTAGTCCGTTAACTCCGTCAAACTTTA 1313
VvERF82      TGGCGG------CGGCCAGTCATG------GGGTTAGTCCGTTAACTCCGTCAAACTTTA 1310
             ******      *  * *** **       ** ** **** ****  ** ***** *  *

VvERF83      GGGCCGTGTGGGAGGAGAGAGAGATGGAAGGAATATTCAAGTTGCCACCGTTAACGCCGT 1369
VvERF85      GGGCCGTGTGGGAGGAGAGAGAGATGGAAGGAATATTCAAGTTGCCACCGTTAACGCCGT 1370
VvERF80      GGGCCGGCTGGGAGGAGAGAGAGATGGAGGGAATATTCCATCTGCCACCGTTAACGCCGT 1373
VvERF82      GGGCCAGCTGGGAGGAGAGAGAGATGGAGGGAATATTCCATCTGCCACCGTTAACGCCGT 1370
             *****   ******************** ********* *  ******************

VvERF83      TATCACCACATCCTTGGTTAGGATATTCTCAGCTTATAGTTTGAG--GAGAGGGTGTGCT 1427
VvERF85      TATCACCACATCCTTGGTTAGGATATTCTCAGCTTATAGTTTGAG--GAGAGGGTTTGCC 1428
VvERF80      TATCACCCCATCCTTGGATAGCATATTCTCAGCTTATAGTTTGAATCAAAGGAGTTTACC 1433
VvERF82      TATCACCCCATCCTTGGATAGCATATTCTCAGCTTATAGTTTGAATCAAAGGAGTTTACC 1430
             ******* ********* *** **********************    *  * ** * *

VvERF83      TTGAATGTAAATATTGAATTTGTTC---ATTCATTTAATAATTTATATCTTTATACTTTC 1484
VvERF85      TTAAATGTAAATATTGAATTTGTTC---ATTCGTTTAATAATTTATATCTTTTTACTT-- 1483
VvERF80      TT-ACCAAAGACATTGAAATATTACTAGAATTCTTACATATTCTATGAACA-ATATTGAA 1491
VvERF82      TT-ACCAAAGACATTGAAATATTACTAGAATTCTTACATATTCTATGAACA-ATATTGAA 1488
             ** *    * * ****** *  * *   * *  **  *** * ***       ** *

VvERF83      GGTTACGGTTGCCATCAAATTCCATCTTCACAAATATACTTAATTGAGAGTAACCCATTT 1544
VvERF85      ----ATGGTTGCCCTCCAAATCAATATTCACAAAGATACTTCAATGAGAGTAGCTGATAT 1539
VvERF80      TTTGTTTATATCTCACACTGTTCCTTTTTTGTCGTCCTATAAATCAATCATTACAAAAAT 1551
VvERF82      TTTGTTTATATCTCACACTGTTCCTTTTT-GCCGTCCTCTAAATCAATCATTACAAAAAT 1547
                     *  *   *    *   * **           *  *    *  *  *  *  *

VvERF83      A-TATATATAAT-CGT--TGTACTAGTGTGGTGGAGACTCACAATGG-GTTAAAGGGAGT 1599
VvERF85      AGTACCTATAGG-CGCACAAAATCATCTCAGCTCAAAATCAGGACTA-TAAAAAGGACAT 1597
VvERF80      ACTTCCGCCAGGGCTTAAAATACTAGC-TGATGCACATAGCTGACAACTTTATAAGATGA 1610
VvERF82      ACTTCGGCCAGGGCTTAAAATACTAGC-TGATGCACATAGGTGACAACTTTATAAGATGA 1606
               * *      *   *       *  *         * *      *       * * *

VVERF83      TGAAACTAAGTTTGCTTATTTTATTTC----CTATGTATATGAGACCCAGTCAGACCTGT 1655
VVERF85      TCCGAACCTCCTGGCACAAATCATCTCAAAATCAGGACCATAAAAAAAAGGACCTCCCGA 1657
VVERF80      TATAAGTTCTTTTTTTTCTTTTTTCTT----TTTAATTTATCAAATTAATTAATTAATAA 1666
VVERF82      TATAAGTTTTTTTTTTTTTTTTTTT----------AATTTATCAAATTAATTAATTAATAA 1656
             *   *      *        *  *               ** * *   *

VVERF83      GGTAAAGGGTGCAATAGATTTTTTTAAT-GAATCAATCATTTAATCGAAAAAGTTATTCA 1714
VVERF85      ACCTCTTGGACTTACAGGATGATCTGAC-TAATTGACC-TATGTCTAAAAAGACGAGTCC 1715
VVERF80      TAAAATAACAACAACAA--TATTTTAATAGGGTTGATAGAGTAATTTTTA--GCTGGTCC 1722
VVERF82      TAAAATAACAACAACAA--TATTTTAATAGGGTTGATAGAGTAATTTTTA--GCTGGTCC 1712
                          * *   *  * * *     *  *     *       *       **

VVERF83      ATCTAATGCGGGAATCTAGTGCTTAGATTCTCCAATTAAACAAAT--GCGTGATA-TACT 1771
VVERF85      AACTACCCTTGTCCTCATGTGCCCAAAAGT--CAATAAAGTCTATTGACATGCTA-TAAA 1772
VVERF80      AT-TAGCAT-GCATGCATGCATGCAAGTGGAGTATGAAATGGCATAGGTATCTTAGTAGT 1780
VVERF82      AT-TAGCAT-GCATGCATGCATGCAAGTGGAGTATGAAATGGCATAGGTATCTTAGTAGT 1770
             *  **     *    *  *     *        *   **    **     *  ** **

VvERF83      CATTGCCCTCGACTGGGTTAATTTAATCAGGGGAGGATGCTGTGTCATGATAAGCATATA 1831
VvERF85      AAGTTTGCTAACCTA--TTACAAAAGTCACTCCTTCATACTACTTCACAAGGAAAA-ATG 1829
VvERF80      GAACTTGGT--ATTATTATTATTTTTTTAAAAATGAAAATTATTTAAAAAGGAGAATATT 1838
VvERF82      GAACTTGGT--ATTATTATTATTTTTTTAAAAATGAAAATTATTTAAAAAGGAGAATATT 1828
              *      *    *    *       * *       *   *   * *  *  *  * **

VvERF83      CCCGTTTTCTATTTGTGTTTTGTCTCTTTGGTATATTCTAAAGAAACTACTTGTCTGCTT 1891
VvERF85      ACTATAAAAGGGGAGTCTCTT-CCTCCTCGAGGGATCCT-------CTCTTTGCCTCTAA 1881
VvERF80      TTTAAATAAAGACAATTAAATTTGGATATAATAGATCAAAA-----CTATGAAATGGCAA 1893
VvERF82      TTTAAATAAAGACAATTAAATTTGGATATAATAGATCAAAA-----CTATGAAATGGCAA 1883
                            *    *             **          **
```

```
VvERF83    TGTGGGAGATAAAAAATTCATACTCATGCATGGGCCCTCCACATATGTA--TATACAAAC 1949
VvERF85    AAGGAAATGCAAAAAATGTATCTTTGTATCCAAAAATGATGGAATAAAA--TATTCAATA 1939
VvERF80    AGGAAGAAAAGTTGATACTAATTTTAGGCCTACAATGGGGAAACCTAAAATTATGGGATC 1953
VvERF82    AGGAAGAAAAGTTGATACTAATTTTAGGCCTACAATGGGGAAACCTAAAATTATGGGATC 1943
                         *       *    *   *                  *     *  ***   *

VvERF83    CCAACTCTTTGTGA---TAGATATTTTATTATT-TTATATATTATTAAAAATTTA----- 2000
VvERF85    TTTTCCTTAAAGAATTTTAGCATCTTCATAATTATTGTACATTGTGAAGAAGCTTCCACT 1999
VvERF80    ATAATTTATTCTGATCTAAGAGTCTTCAATATC-TTCAATATTCACCC------------ 2000
VvERF82    ATAATTTATTCTGATCTAAGAGTCTTCAATATC-TTCAATATTCACCCCCATAAGATG-- 2000
                        *    **    ** *  **  **  * ***
```
