# Supplementary material for: Genomic and transcriptomic analysis of the AP2/ERF superfamily in Vitis vinifera
Source: BMC Genomics. 2010 Dec 20;11:719. doi: 10.1186/1471-2164-11-719 (PMC3022922; doi:10.1186/1471-2164-11-719)
Supplement: Additional file 9 — Correlation of the expression of group-IX ERFs and their putative target genes. Correlation of the expression of group IX-ERFs and their putative target genes. 1,4-D-glucanase (CF212592), CHIV1 (AY137377), 1,3-Glucanase3 (TC62849), 1,3-Glucanase1 (CF605842), thaumatin-like3 (TC56535) and CHIV2 (TC64563). The Pearson Correlation coefficient (PC) was calculated using the expression values provided by Deluc et al. (2007) [31]. [file 1471-2164-11-719-S9.PDF]

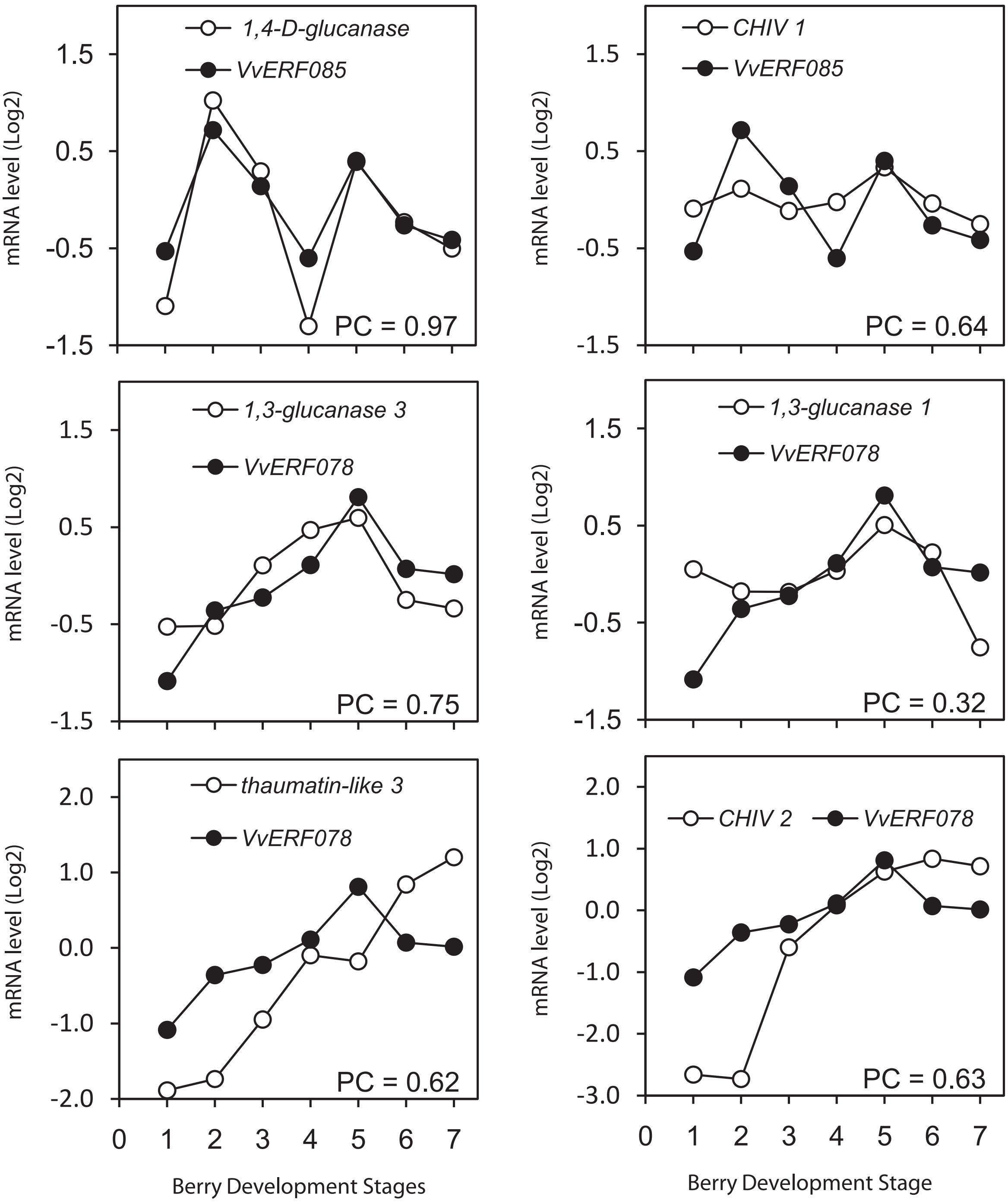

Additional **Figure S9**. Correlation of the expression of group IX-ERFs and their putative target genes. 1,4- D-glucanase (CF212592), CHIV1 (AY137377), 1,3-Glucanase3 (TC62849), 1,3-Glucanase1 (CF605842), thaumatin-like3 (TC56535) and CHIV2 (TC64563). The Pearson Correlation coefficient (PC) was calculated using the expression values provided by Deluc et al. (2007).
